# Acute Respiratory Distress Syndrome in Hospital-Acquired/Ventilator-Associated Pneumonia

**DOI:** 10.3390/medsci14050514

**Published:** 2026-08-25

**Authors:** Davide Calabretta, Claudia Accetturo, Antoni Torres

**Affiliations:** 1 Applied Research in Respiratory Infections and Critical Illness, Institut d’Investigacions Biomèdiques August Pi i Sunyer (IDIBAPS), 08036 Barcelona, Spain; 2 School of Medicine and Health Sciences, University of Barcelona, 08036 Barcelona, Spain; 3 Department of Anesthesia and Critical Care, ASST Ovest Milanese Ospedale Civile di Legnano, 20025 Milan, Italy; 4 Department of Pathophysiology and Transplantation, Università degli Studi di Milano, 20122 Milan, Italy; 5 Ciber de Enfermedades Respiratorias (Ciberes) Barcelona, 08025 Barcelona, Spain

**Keywords:** hospital-acquired pneumonia, ventilator-associated pneumonia, acute respiratory distress syndrome, ICU, inflammation, pulmonary microbiome

## Abstract

Acute respiratory distress syndrome (ARDS) represents a major cause of morbidity and mortality in critically ill patients and is most frequently triggered by severe respiratory infections, including nosocomial pneumonia. Hospital-acquired and ventilator-associated pneumonia (HAP/VAP) are highly prevalent in intensive care units and share overlapping pathophysiological mechanisms with ARDS. Despite this close interrelationship, the proportion of patients with nosocomial pneumonia who subsequently develop ARDS remains poorly defined, underscoring the need to identify potential predisposing factors and improve early recognition of patients at risk. While the development of pneumonia in patients with an established diagnosis of ARDS has been extensively investigated, limited data are available on patients who develop ARDS as a complication of HAP/VAP. This narrative review summarizes the limited direct evidence on this topic and discusses potential clinical characteristics, risk factors, and predictive tools, while considering indirect evidence from CAP and broader ARDS populations.

## 1. Introduction

Acute respiratory distress syndrome (ARDS) is a life-threatening clinical syndrome characterized by acute hypoxemic respiratory failure, bilateral pulmonary opacities, and non-cardiogenic pulmonary edema [1]. It arises from a dysregulated immune response driven by a cascade of pro-inflammatory cytokines, ultimately resulting in loss of aerated lung volume, increased alveolar–capillary permeability, and augmented pulmonary shunt and dead space [2,3]. ARDS can be triggered by a wide range of insults, but the most common cause is severe respiratory infection, particularly community-acquired (CAP) and nosocomial pneumonia, including both hospital-acquired pneumonia (HAP) and ventilator-associated pneumonia (VAP) [4,5,6]. Nosocomial pneumonias are the second most common cause of hospital-acquired infections overall, yet they are the leading cause of infection among critically ill patients, resulting in a heavy burden in terms of mortality and costs for the healthcare system [7].

Sharing multiple pathophysiological mechanisms and similar risk factors, nosocomial pneumonia and ARDS are closely interconnected and frequently associated in critically ill patients [8].

While nosocomial pneumonia may complicate established ARDS, the proportion of patients with HAP/VAP who subsequently develop ARDS remains poorly defined. Identifying patients potentially at increased risk may nevertheless be clinically relevant for early recognition and monitoring.

The processes leading to the development of nosocomial pneumonia (and especially VAP) in individuals with ARDS have been extensively characterized over the years [5,9,10,11] but far less data are available regarding patients with nosocomial pneumonia who subsequently develop ARDS [12].

This narrative review focuses on the progression from HAP/VAP to ARDS, summarizing shared pathophysiological mechanisms and clinical features associated with increased risk. Because direct evidence in HAP/VAP populations is limited, selected indirect evidence from CAP and broader ARDS populations was also considered and explicitly distinguished from direct evidence throughout the review. The bidirectional relationship between pneumonia and ARDS is discussed to contextualize the comparatively well-characterized occurrence of HAP/VAP in patients with established ARDS and the substantially less studied progression from HAP/VAP to ARDS. The literature was identified primarily through PubMed/MEDLINE searches, supplemented by selected articles retrieved from DOAJ, and included studies published or indexed up to 28 February 2026.

## 2. Respiratory Infections and ARDS: A Complex Relationship

The relationship between respiratory infections and ARDS is bidirectional, with each condition potentially favoring the onset of the other. In the context of this review, this relationship is relevant because the two temporal sequences are supported by markedly different levels of evidence: HAP/VAP occurring in patients with established ARDS has been more extensively characterized, whereas the development of ARDS after HAP/VAP remains insufficiently studied.

In the international LUNG SAFE cohort, pneumonia was identified as a risk factor for ARDS in 59.4% of patients, making it the most frequently reported precipitating condition. Severe pulmonary infection may contribute to ARDS through dysregulated and self-amplifying inflammatory responses, resulting in alveolar–capillary barrier disruption, pulmonary edema, and diffuse alveolar injury [13].

*Streptococcus pneumoniae* is the most common pathogen isolated in patients with CAP [14]; however, the incidence of ARDS does not appear to be higher in pneumococcal pneumonia than in other bacterial etiologies and species such as *Mycoplasma pneumoniae*, *Pseudomonas aeruginosa*, and *Staphylococcus aureus* are frequently involved in cases that progress to ARDS [15]. Furthermore, viruses are an increasing cause of pneumonia that can lead to ARDS, including seasonal agents such as adenovirus, metapneumovirus, respiratory syncytial virus, and influenza viruses [16].

Influenza is the most frequent seasonal viral cause of pneumonia and is also responsible for the development of epidemics and pandemics, such as the 2009 H1N1 outbreak, during which progression to ARDS and associated mortality were markedly increased. A similar trend was also observed with coronaviruses [17,18] and the recent SARS-CoV-2 pandemic, in which nearly all of the most severe cases admitted to the ICU met the Berlin criteria for the definition of ARDS [2].

The evidence supporting this reverse sequence is substantially more developed than the evidence concerning ARDS developing after HAP/VAP. On the other hand, the onset of pneumonia is also facilitated by ARDS, and the conditions of intubation and prolonged mechanical ventilation ensure that VAP represents one of the most frequent complications, with an estimated incidence of 20–40% depending on the case series [5].

**Figure 1:**
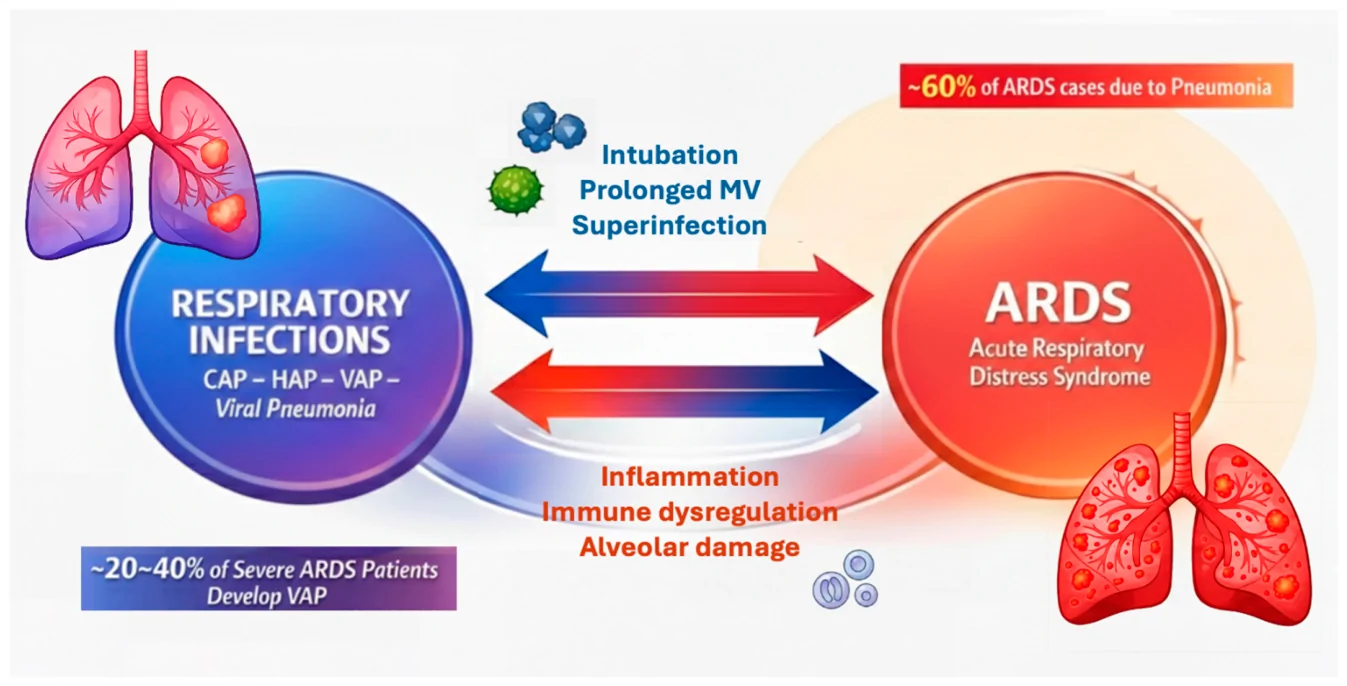
A graphic representation of the bidirectional relationship between respiratory infections and ARDS. The majority of ARDS cases are secondary to pneumonia and develop as a result of dysregulated inflammation and an immune response leading to diffuse alveolar damage (DAD). Conversely, in patients with ARDS, the impairment of host defense mechanisms due to intubation and prolonged mechanical ventilation facilitates superinfection pathways, which in turn drive the development of pneumonia. Abbreviations: CAP = Community-acquired pneumonia HAP = Hospital-acquired pneumonia; VAP = Ventilator-associated pneumonia; MV = Mechanical ventilation.

Figure 1 provides a concise representation of the bidirectional relationship between respiratory infections and ARDS, illustrating the different pathophysiological mechanisms that link the two conditions.

There is a substantial diagnostic difficulty in identifying ventilator-associated pneumonia complicating ARDS, as the two conditions share overlapping clinical and radiological features: hypoxemia, impaired gas exchange, and similar lung infiltrates make it challenging to differentiate superimposed infection from ARDS progression [6].

Systemic signs such as fever and leukocytosis do not reliably discriminate infectious from sterile inflammation, and imaging also has important limitations: CT provides higher sensitivity and specificity than chest radiography but is often difficult to perform in critically ill patients. Finally, bilateral infiltrates required for ARDS diagnosis complicate the detection of new or worsening opacities needed to confirm VAP [19]. In this context, microbiological sampling, via tracheal aspirate (TAS) or broncho-alveolar lavage (BAL), is crucial for guiding diagnosis, although the interpretation of results is complicated by the frequent colonization of proximal airways in intubated patients, which may mimic infection. BAL, by sampling distal airways, improves discrimination between colonization and superinfection and reduces false positives compared with endotracheal aspirate, but remains prone to false negatives during antibiotic therapy, especially in infection-related ARDS [7].

New diagnostic tools are emerging in this setting, including blood biomarkers, molecular pathogen identification techniques and cytokine levels assessment, but currently lack sufficient evidence to influence antibiotic prescription [20].

Although the occurrence of VAP in patients with established ARDS has been extensively investigated, direct evidence concerning patients with HAP/VAP who subsequently develop ARDS remains markedly limited.

Several factors may explain this paucity of evidence. First, most studies evaluating pneumonia-associated ARDS predominantly include patients with community-acquired pneumonia, whereas HAP and VAP are rarely analyzed as distinct entities. Furthermore, conducting prospective studies in critically ill patients is inherently challenging because of disease heterogeneity, multiple competing risk factors, and the complex interplay between infection, mechanical ventilation, and host immune responses [21,22]. The diagnostic overlap among severe pneumonia, sepsis-associated lung injury, and ARDS may further complicate patient classification, while current ARDS cohorts often report pneumonia as a single etiological category, potentially underestimating the specific contribution of HAP and VAP as triggers of ARDS.

Within this limited evidence base, Barbeta et al. conducted the main study specifically addressing progression from VAP to ARDS. Among 301 patients with VAP, 13.6% developed ARDS. However, this finding derives from a single observational study and should not be generalized to all VAP patients or to HAP populations. Data specifically addressing HAP are lacking, while larger ARDS datasets generally classify pneumonia as a broad etiological category without consistently distinguishing CAP from HAP or VAP [13].

The majority of pneumonia cases that arise in the ICU, however, are HAP/VAP, and clinical practice suggests that the nosocomial etiology represents a relevant slice of the infections that evolve into ARDS, especially when it presents with marked severity or is sustained by multidrug-resistant microbial strains that are not promptly treated with effective antibiotic therapy [23].

Targeted studies in this population would be valuable to better delineate the clinical characteristics and risk factors associated with progression to ARDS. In the absence of validated criteria for pre-ARDS risk stratification in patients with HAP/VAP, the present review focuses on prevention and on the early recognition of patients at increased risk of progression.

## 3. Shared Pathways and Progression from Pneumonia to ARDS

As previously mentioned, the close relationship between pneumonia and ARDS is attributable to shared pathogenic pathways that tightly link the two conditions. Specifically, the two elements playing a key role in the evolution of the infectious process towards ARDS development are (1) inflammatory dysregulation and (2) alterations in the pulmonary microbiome.

**(1)**  **The role of lung inflammation**

In patients who develop pneumonia, the presence of pathogens leads to the establishment of an inflammatory cascade involving both the organism’s innate and adaptive immunity.

This response begins with the activation of neutrophils, macrophages, and respiratory epithelial cells, which results in the production of chemokines such as CXCL2, CXCL5, and CCL2, expression of growth factors such as GM-CSF (granulocyte–monocyte colony stimulating factor), and cytokines IL-1 β, IL-6, and TNF-α [24]. These events recruit and activate more neutrophils, macrophages, eosinophils, basophils, dendritic cells, as well as NKs at sites of infection and injury, leading to a positive feedback phenomenon that involves the alteration of cell death pathways and the release of a variety of endogenous molecules collectively named pathogen-associated molecular patterns [25,26] (PAMPs) and damage-associated molecular patterns (DAMPs) [27].

These inflammatory mediators are recognized by specific receptors such as TLRs (Toll-like receptors), RLRs (retinoic acid-inducible gene-I-like receptors) and NLRs (NOD-like receptors) that mediate the intracellular activation of the inflammasome [28]. This structure plays a fundamental role in the positive feedback mechanism of the innate immune response to infection, also triggering the release of neutrophil extracellular traps (NETs) [29,30]. These filamentous extracellular structures, composed of chromatin, histones, and neutrophil antimicrobial agents, are primarily generated to counteract microbial infection. In the case of a dysregulated inflammatory response, however, the phenomenon known as NETosis occurs, in which the loss of control over the process ultimately further amplifies the inflammatory cascade, contributing decisively to the endothelial and alveolar epithelial damage that underlies ARDS [31,32].

Several of these inflammatory mediators have been proposed as potential biomarkers to reflect distinct components of the dysregulated host response in ARDS and severe pneumonia. In particular, elevated levels of IL-6, angiopoietin-2, and sRAGE have been associated with inflammatory burden, endothelial and epithelial injury, disease severity, and adverse outcomes in ARDS [25,26]. Biomarkers related to neutrophil activation and NET formation, including circulating cell-free DNA, have also been explored as indicators of excessive innate immune activation and tissue injury.

However, these biological indicators remain purely exploratory, and none of these biomarkers has yet been validated for routine clinical prediction or risk stratification of ARDS development, particularly in patients with HAP/VAP [33]. Therefore, this mechanistic discussion is intended to highlight promising avenues for future research rather than to support current routine clinical decision-making. Further studies are needed to determine whether biomarker-based strategies can eventually improve early risk stratification and support personalized management in this population.

The interplay between these inflammatory processes extends beyond the host response, involving complex interactions between the lung environment and the resident microbial community. Alterations in the lung microbiota, including microbial depletion and loss of ecological balance, may further contribute to the disruption of homeostasis and the persistence of a pro-inflammatory state [8].

**(2)**  **The role of pulmonary dysbiosis**

Although long considered sterile, it is now recognized that the lungs are physiologically colonized by a resident respiratory microbiome. This microbial flora, which is well characterized, possesses a fundamental protective role for the lung, and an alteration in its composition is defined as pulmonary dysbiosis [34].

In the critically ill patient, phenomena such as antibiotic exposure, mechanical ventilation, microbial translocation (due to microaspiration or hematogenous transposition), and the alteration of host defense mechanisms like impaired cough reflex, immunity, and mucociliary clearance can lead to these alterations, depleting the microbiome and compromising its protective role [35].

Studies on VAP have indeed demonstrated that intubated patients who develop pneumonia are characterized by the following [36,37]:

- Reduced richness (number of different taxa) and evenness (distribution of taxa abundance) within the commensal microbial community that constitutes the pulmonary microbiota (loss of alpha-diversity).
- Reduced regional differences between the composition of the lung microbiome and that of other high-biomass sites, such as the digestive tract (loss of beta-diversity).
- Increased microbial abundance associated with depletion of protective bacterial strains (*Streptococcus* spp., *Enterococcus* spp., *Lactobacillus* spp., and *Prevotella* spp.) in favor of dominant pathogenic species.

This loss of biodiversity associated with the reduction in commensal species probably represents the fundamental mechanism that leads to the onset of pneumonia in the intubated patient. However, recent literature data show that the increase in gut-associated bacteria (*Pasteurellaceae* spp. and especially *Enterobacteriaceae* spp.) and lung microbial DNA are also associated with ARDS [38,39].

A recent study demonstrated that the development of ARDS in the intubated patient is not associated with baseline composition, but rather with lung bacterial composition at 48 h, suggesting that early shifts in bacterial composition may play a role in the progression of the disease [40,41].

**Figure 2:**
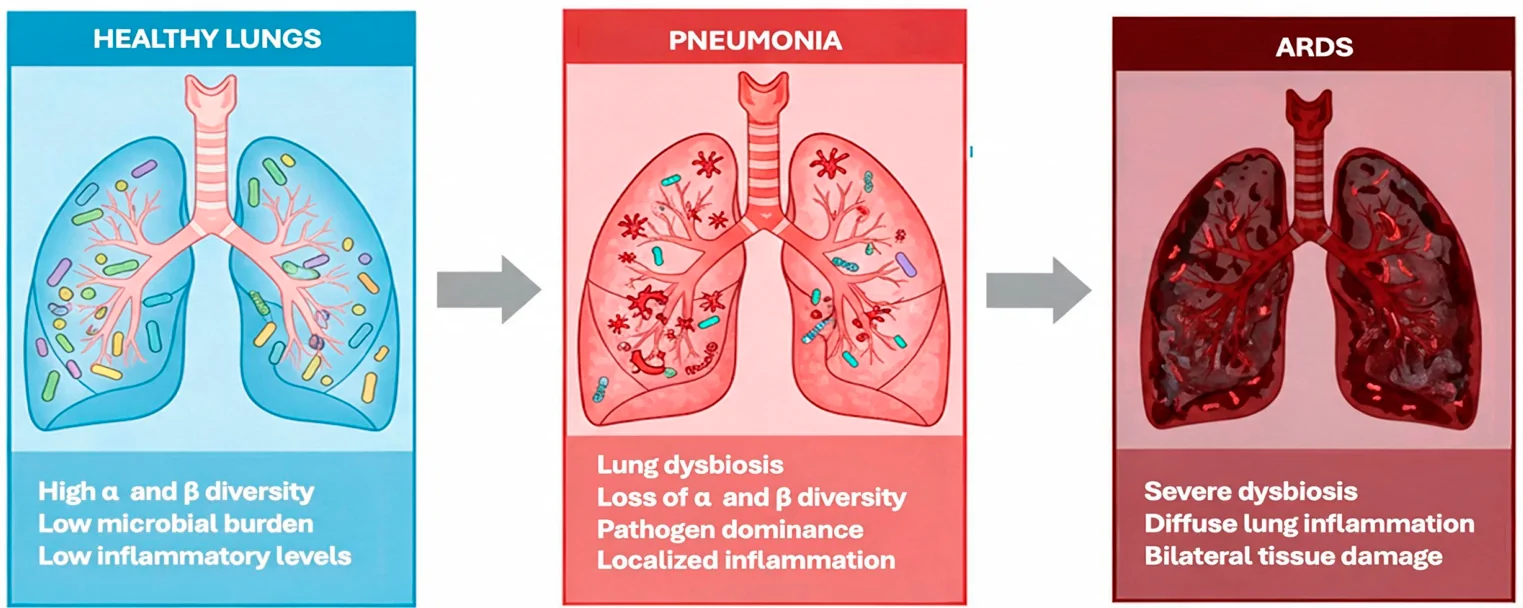
A schematic model illustrating the progression from a healthy lung to pneumonia and eventually ARDS (acute respiratory distress syndrome). This representation highlights the potential interplay between pulmonary microbiota alterations and dysregulated inflammation, which may contribute to a vicious cycle leading to ARDS development.

As summarized in Figure 2, the presence of a positive feedback loop of inflammation and dysbiosis therefore appears to be a fundamental factor within the process that leads to the onset of ARDS in patients with pneumonia. Nevertheless, data on this matter are still scarce, and further future studies will be necessary to define the causal links within this complex relationship.

## 4. Characteristics of Patients with Nosocomial Pneumonia Who Develop ARDS

As discussed above, pneumonia is a frequent risk factor for ARDS in critically ill patients. However, the proportion of patients with HAP/VAP who subsequently develop ARDS remains poorly defined, and direct evidence in this population is scarce.

The only study specifically addressing this population is a single-center observational study that includes exclusively critically ill patients with VAP [12]. In this work, patients were stratified according to the subsequent development of ARDS, and risk factors associated with the development of this condition were described.

Among patients with VAP (enrolled within a cohort of 800 intubated subjects), the ones who developed ARDS were, on average, younger and showed a lower prevalence of chronic obstructive pulmonary disease (COPD) and a higher prevalence of chronic liver disease. At ICU admission, these patients had lower SAPS II scores compared with controls; however, at the time of VAP diagnosis, SOFA score was significantly higher in patients who developed ARDS, suggesting greater clinical severity during the infectious phase. In both groups, *Pseudomonas aeruginosa* was the most frequently isolated pathogen, with no significant differences in the prevalence of multidrug-resistant (MDR) strains or in the appropriateness of empirical antibiotic therapy. Based on the PaO_2_/FiO_2_ ratio, most patients who developed ARDS were classified as having moderate severity, while the remaining cases equally distributed between mild and severe forms. Mechanical ventilation parameters did not differ significantly between groups, with the exception of a higher respiratory rate required in patients with ARDS. The development of ARDS as a complication of VAP was not associated with significant differences in the primary outcome of 90-day mortality, nor in secondary outcomes.

Although based on a relatively limited sample size, the study by Barbeta et al. represents a unique contribution to the literature, as it specifically investigates the progression from VAP to ARDS. The findings reported in this paper should, however, be interpreted with caution.

The studies discussed below derive from cohorts of patients with CAP or general mechanically ventilated populations and are therefore presented as indirect evidence. They provide clinical and biological context in the absence of dedicated HAP/VAP cohorts, but should not be interpreted as validated HAP/VAP-specific risk factors.

A study by Cilloniz et al. [15], assessing the impact of ARDS among patients hospitalized with CAP, demonstrated that higher SOFA scores and previous antibiotic use were independent predictors for ARDS among ventilated patients, whereas corticosteroid use appeared to be a protective factor. Notably, as in the case of Barbeta et al., the diagnosis of ARDS was not correlated with any specific pneumonia etiology nor with increased 30-day mortality among mechanically ventilated patients.

A large multicenter observational study conducted in mechanically ventilated patients who developed ARDS found that higher airway pressures at the onset of mechanical ventilation, the need for higher levels of PEEP, and a higher body mass index were associated with the development of ARDS [42].

Another paper evaluating patients with ARDS developing in the context of pneumonia reported that inappropriate initial antimicrobial therapy and blood transfusion independently predicted the development of acute lung injury, regardless of the causative pathogen [43].

Finally, in 2014, Ahmed et al. [44] considered preventable hospital exposures and found that also hospital-acquired aspiration and volume of administered fluids were related with higher risk of ARDS.

These findings further underscore the complex pathogenesis underlying the development of ARDS in patients with pneumonia and reflect the marked heterogeneity of this population. For this reason, several tools have been developed to predict the risk of ARDS. Among these, the most widely used is the Lung Injury Prediction Score (LIPS) [45], reported in Table 1.

This predictive model incorporates both predisposing conditions and risk modifiers, assigning a variable score according to the presence of each factor. LIPS has demonstrated good predictive performance and has been adapted for multiple patient subpopulations, including in combination with biomarkers or other predictive tools [46,47]. However, its specificity remains limited, particularly in critically ill patients, and it has not been specifically developed or validated for patients with HAP/VAP.

Nevertheless, only a small proportion of patients with these risk factors ultimately progress to ARDS, which restricts the practical implementation of these tools in clinical practice. Consequently, in recent years, several authors have explored machine learning-based models to improve prediction of ARDS development in selected patient populations [48,49,50,51].

Although these models hold promise for providing clinicians with improved tools to identify patients at risk, they still require rigorous external validation and careful assessment of calibration and discrimination before widespread implementation. Furthermore, it remains uncertain whether machine learning approaches provide clinically meaningful improvements over traditional prediction models, particularly in patients with nosocomial pneumonia.

## 5. Prevention and Management of Secondary ARDS in Nosocomial Pneumonia

Identifying patients with HAP/VAP at high risk of developing ARDS represents a fundamental step in disease management, as numerous preventive and therapeutic measures can be implemented to limit its progression. Although a preventive approach to ARDS specifically for patients with HAP/VAP has not been extensively studied in the literature, Table 2 aims to summarize the main preventive strategies that can be adopted to reduce its incidence in this population. The present table adapts the ESICM guideline recommendations [52] to the management of HAP/VAP cases, with the aim of providing a clinically useful tool for the treatment of high-risk patients.

To begin with, the management of pneumonia requires the administration of early appropriate antimicrobial treatment, and failure to administer this treatment has been correlated with an increased incidence of ARDS and other negative outcomes [44,53]. Current guidelines for HAP/VAP [23,54] recommend broad-spectrum empirical antibiotic treatment based on the patient’s risk factors at the time of pneumonia diagnosis, which should subsequently be followed by a targeted treatment based on the identified causative pathogen. However, the development of rapid microbiological techniques is challenging this principle and has been shown to achieve the prescription of appropriate treatment in a shorter time. Nevertheless, a clinical benefit of this approach in terms of patient outcomes has not yet been extensively demonstrated [55,56].

Despite the use of corticosteroids having been widely adopted in severe CAP [57] and extensively used in severe forms of COVID-19 after the publication of data from the RECOVERY trial [58], their use remains controversial and should generally be avoided in the treatment of HAP/VAP unless there is concomitant septic shock or ARDS [59].

In the latter case, the employment of early dexamethasone has proven effective in controlling inflammation and counteracting disease progression in moderate and severe cases of ARDS [60]. Notwithstanding this, recent meta-analyses have demonstrated significant heterogeneity in study results regarding the molecule, dosage, and disease etiology considered, and current data do not support their routine use in ARDS since observational studies and RCTs have produced conflicting results. Therefore, the use of corticosteroids for the prevention and treatment of ARDS should not be routinely considered and should only be evaluated in selected cases [22,61].

The recognition of the risk of ventilator-induced lung injury (VILI) has led to the development of lung-protective ventilation strategies but conflicting evidence has been produced over the years regarding the optimal ventilatory strategy in ARDS patients [62]. Nevertheless—according to the recent recommendations of the 2023 ESICM guidelines—patients should be ventilated with an appropriate PEEP (positive end-expiratory pressure) aimed at maintaining an open lung, and with a tidal volume ranging between 4 and 8 mL/kg of predicted body weight. Recruitment maneuvers should only be performed, when necessary, while avoiding long high-pressure maneuvers (P ≥ 35 cmH2O for more than one minute) which have been associated with a higher incidence of clinical complications [52,63,64].

Continuous infusion of neuromuscular blocking agents (curare) should be avoided to prevent ICU-acquired neuromuscular weakness and to allow for frequent neurological windows to facilitate sedation titration and a more rapid resumption of spontaneous muscular activity [65,66]. Prone positioning should be performed in moderate-to-severe forms (P/F ≤ 150 despite optimized ventilatory settings) in order to improve clinical outcomes and patients who meet the EOLIA criteria for veno-venous extra-corporeal membrane oxygenation (vv-ECMO) appear to benefit from this type of treatment and should be centralized to referral centers capable of offering this life support option [67].

In HAP/VAP cases with a high risk of progression to ARDS, fluid management must be conservative, aiming to restore adequate tissue oxygen delivery without increasing non-cardiogenic pulmonary edema [68]. Similarly, the use of blood transfusions must be particularly cautious, employing restrictive thresholds (Hb ≤ 7.0 g/dL) because the use of liberal transfusion thresholds has not resulted in clinical benefits and is associated with the onset of TRALI (transfusion-related acute lung injury), the occurrence of which may mimic or exacerbate the ARDS presentation [69].

Finally, considering patients with HAP and respiratory failure, it is important to underline that the use of a High-Flow Nasal Cannula (HFNC), despite not demonstrating a benefit in terms of mortality, has been shown to be effective in reducing the incidence of intubation when compared with low-flow oxygen therapy, and thus may be considered for the prevention of ARDS in these high-risk patients [52,70,71].

**Table 2:** Strategies for the prevention and management of ARDS in patients with HAP/VAP. Adapted from ESICM recommendations for the management of ARDS and contextualized for the HAP/VAP population.

Intervention Category	Specific Strategy	Recommendations	Rationale and Evidence
Antimicrobial Therapy	Appropriate antimicrobial treatment	Broad-spectrum empirical therapy → targeted therapy as soon as possible	Inappropriate antimicrobial treatment is associated with increased ARDS incidence and worse outcomes [23,53,54]
	Rapid microbiology	Faster pathogen identification	May allow for earlier appropriate treatment; however, their clinical benefit remains not fully established [55,56].
Corticosteroids	Dexamethasone in ARDS	Early use in moderate-to-severe ARDS	Effective in controlling inflammation; conflicting results in meta-analyses [57,58,60]
	In HAP/VAP	Avoid unless septic shock or ARDS present	Not routinely recommended; significant heterogeneity in studies [59]
Mechanical Ventilation	Tidal volume	4–8 mL/kg predicted body weight	Lung-protective ventilation strategies have been shown to reduce the incidence of ARDS in mechanically ventilated patients [52].
	PEEP	Appropriate level to maintain open lung	As comparisons between different protocols have not demonstrated clear superiority, an individualized approach is recommended [52]
	Recruitment maneuvers	Only when necessary; avoid P ≥ 35 cmH2O > 1 min	several strategies have been proposed; prolonged high-pressure maneuvers are associated with potential complications and should be avoided [52]
	Neuromuscular blockade	Avoid continuous infusion, if possible	Prevents ICU-acquired weakness; allows for neurological assessment [65,66]
	Prone positioning	Moderate-to-severe ARDS (P/F ≤ 150)	The use may be considered in selected patients with moderate–severe ARDS [52,64]
	vv-ECMO	Patients meeting EOLIA criteria	Should be considered in case of diagnosis of severe ARDS and presence of the criteria. Referral to specialized centers [67]
Fluid Management	Conservative strategy	Restore tissue O_2_ delivery without increasing edema	Conservative fluid management has been shown to reduce pulmonary edema [68]
Transfusion	Restrictive threshold	Hb ≤ 7.0 g/dL	Liberal strategies have not demonstrated benefit and may increase the risk of TRALI [69]
Respiratory Support	High-Flow Nasal Cannula	For HAP with respiratory failure	Effective in reducing intubation rates compared with conventional low-flow oxygen therapy [70,71]

Definition of abbreviations: ARDS = acute respiratory distress syndrome; HAP = hospital-acquired pneumonia; VAP = ventilator-associated pneumonia; PEEP = positive end-expiratory pressure; ECMO = Veno-venous extra-corporeal membrane oxygenation; Hb = hemoglobin; TRALI = transfusion-related acute lung injury.

## 6. Knowledge Gap and Future Directions

Despite the well-established association between pneumonia and ARDS, the specific clinical and biological characteristics of patients with HAP/VAP who subsequently develop ARDS remain insufficiently characterized. Current evidence is limited, partly because pneumonia-associated ARDS is frequently considered a single entity, despite relevant differences between community-acquired pneumonia (CAP), hospital-acquired pneumonia (HAP), and ventilator-associated pneumonia (VAP) in terms of microbiology, host characteristics, timing of infection, and exposure to mechanical ventilation. A more refined characterization of these populations may allow for the identification of distinct phenotypes and contribute to the development of tailored management strategies.

Future research should prioritize dedicated multicenter prospective cohorts incorporating standardized definitions of both pneumonia and ARDS. Integrating biomarker-based approaches, including markers of inflammation, epithelial injury, and endothelial dysfunction, may improve early risk stratification and facilitate the identification of patients more likely to benefit from targeted interventions. Furthermore, microbiome-based analyses may provide novel insights into host–microbe interactions and mechanisms driving disease progression. Artificial intelligence and machine learning approaches may also offer promising opportunities by integrating clinical, microbiological, imaging, and biological data to enhance individualized risk prediction.

Ultimately, advancing our understanding of the complex interplay between pathogen factors, host response, and lung injury will be crucial to move toward precision medicine approaches for patients with pneumonia-associated ARDS and improve outcomes in this high-risk population.

## Figures and Tables

**Table 1:** Lung injury prediction score (LIPS) calculation worksheet. A score ≥ 4 has been shown to effectively discriminate the general population from those who go on to develop ARDS. Adapted from Gajic et al. [46] (Am J Respir Crit Care Med Vol 183. pp. 462–470, 2011).

	Lips Points
**Predisposing conditions**	
Shock	2
Aspiration	2
Sepsis	1
Pneumonia	1.5
High-risk surgery *	
● Orthopedic spine	1
● Acute abdomen	2
● Cardiac	2.5
● Aortic vascular	3.5
High-risk trauma	
● Traumatic brain injury	2
● Smoke inhalation	2
● Near drowning	2
● Lung contusion	1.5
● Multiple fractures	1.5
**Risk modifiers**	
Alcohol abuse	1
Obesity (BMI > 30)	1
Hypoalbuminemia	1
Chemotherapy	1
FIO2 > 0.35	2
Tachypnea (RR > 30)	1.5
SpO2 < 95%	1
Acidosis (pH < 7.35)	1.5
Diabetes mellitus	−1

Definition of abbreviations: BMI = body mass index; RR = respiratory rate; SpO2 = oxygen saturation by pulse oximetry. * Add 1.5 points if emergency surgery.

## Data Availability

The original contributions presented in this study are included in the article. Further inquiries can be directed to the corresponding author.
